# Cotton *GhMKK1* Induces the Tolerance of Salt and Drought Stress, and Mediates Defence Responses to Pathogen Infection in Transgenic *Nicotiana benthamiana*

**DOI:** 10.1371/journal.pone.0068503

**Published:** 2013-07-03

**Authors:** Wenjing Lu, Xiaoqian Chu, Yuzhen Li, Chen Wang, Xingqi Guo

**Affiliations:** State Key Laboratory of Crop Biology, College of Life Sciences, Shandong Agricultural University, Taian, Shandong, PR China; New Mexico State University, United States of America

## Abstract

Mitogen-activated protein kinase kinases (MAPKK) mediate a variety of stress responses in plants. So far little is known on the functional role of MAPKKs in cotton. In the present study, *Gossypium hirsutum MKK1* (*GhMKK1*) function was investigated. GhMKK1 protein may activate its specific targets in both the nucleus and cytoplasm. Treatments with salt, drought, and H_2_O_2_ induced the expression of *GhMKK1* and increased the activity of *GhMKK1*, while overexpression of *GhMKK1* in *Nicotiana benthamiana* enhanced its tolerance to salt and drought stresses as determined by many physiological data. Additionally, *GhMKK1* activity was found to up-regulate pathogen-associated biotic stress, and overexpression of *GhMKK1* increased the susceptibility of the transgenic plants to the pathogen *Ralstonia solanacearum* by reducing the expression of *PR* genes. Moreover, *GhMKK1*-overexpressing plants also exhibited an enhanced reactive oxygen species scavenging capability and markedly elevated activities of several antioxidant enzymes. These results indicate that *GhMKK1* is involved in plants defence responses and provide new data to further analyze the function of plant MAPK pathways.

## Introduction

Plants face various harsh terrestrial environmental conditions, including high salinity, drought, and microbial pathogens, through their lifetime. To adapt to these environmental stresses, plants have developed sophisticated signaling machineries associated with regulation of their cellular metabolism. Protein phosphorylation is one of the major mechanisms the cell uses to translate external stimuli into cellular responses, and the mitogen-activated protein kinase (MAPK) cascades are involved in many of these processes.

Generally, MAPK cascades are composed of three kinases: MAPKs are phosphorylated by MAPK kinases (MAPKKs) on the threonine residues located in the activation loop (A-loop) between subdomains VII and VIII of the kinase catalytic domain, and the MAPKKs are activated by another group of serine/threonine protein kinases, the MAPK kinase kinases (MAPKKKs), at a conserved S/TXXXXXS/T motif. Compared to our current knowledge of the 20 identified plant MAPKs and the >60 putative MAPKKKs, there is a distinct lack of knowledge about the functions of the 10 MAPKKs found in *Arabidopsis* [1]. These facts suggest that the cross-talk in the MAPK cascades occurs at the level of MAPKKs, and one MAPKK is likely to be involved in multiple MAPK cascades, carrying out diverse biological functions [2].

A growing body of evidence has shown that MAPKKs display important functions in response to abiotic stressors. In *Arabidopsis*, the activation of MAPKK9 induces ethylene and camalexin biosynthesis and enhances the plant's sensitivity to salt stress [3]. In tobacco plants, the dual-specificity protein kinase SIMKK mediates the salt-induced activation of SIMK [4]. During the generation period, AtMEK1 immunoprecipitated from seedlings subjected to wounding, cold, drought, and high salt stimuli shows an elevated kinase activity towards the kinase-negative AtMPK4. This indicates that AtMEK1 becomes active through phosphorylation and then activates its downstream target AtMPK4 during stress responses in *Arabidopsis* [5]. Kong *et al*. reported that ZmMKK4 is a positive regulator of salt and cold tolerance in maize [6]. In *Nicotiana attenuate*, MKK1 and MKK2 are involved in wound healing and in a specialized response to the lepidopteran herbivore *Manduca sexta* [7]. These reports provide us with insight into the functions of the MAPKKs in plants in response to abiotic stress, but the exact function of these kinases needs to be further explored.

Although a number of studies have focused on how MAPKKs function in response to abiotic stress, the role of MAPKK signaling following biotic stress is also an issue of great interest. Emerging evidence has indicated that MAPKKs may be involved in plant defence following biotic stress. In tobacco, the NtMEK2-SIPK/WIPK cascade appears to control the expression of 3-hydroxy-3-methylglutaryl CoA reductase (HMGR) and L-phenylalanine ammonia lyase (PAL), which are defence genes that encode key enzymes in the phytoalexin and salicylicacid biosynthesis pathways. The NtMEK2-SIPK/WIPK cascade pathway plays a positive role in N gene-mediated resistance, possibly through regulating HR cell death [8], [9]. In the *Arabidopsis* leaf cell system, when the early defence genes induced by flagellin, a complete plant MAPK cascade involving MEKK1-MKK4/MKK5-MPK3/MPK6 and the WRKY22/WRKY29 transcription factors has been shown to be stimulated. All of these proteins functioned downstreams of the flagellin receptor FLS2, which is a leucine-rich-repeat (LRR) receptor kinase [10]. In addition, AtMKK3 positively regulates *PR* gene expression and plays a role in the defence against Pst DC3000 [11]. Recently, Gao *et al*. suggested that *GhNDR1* and *GhMKK2* are required for *Verticillium* resistance based on gene-silencing assays performed in cotton [12]. The cross-talk between the MAPK cascades is very complicated, and many functions of the MAPKKs in response to biotic stress remain largely unknown.

Under various stresses, plants produce reactive oxygen species (ROS), including hydrogen peroxide (H_2_O_2_), superoxide anions (O_2_^−^), and hydroxyl radicals [13]. When plants are under stress, the rate of ROS production is dramatically elevated compared to the low levels observed under optimal growth conditions. An imbalance in ROS concentrations can result in oxidative stress and cause irreversible damage. Previous studies have suggested that the MAPK pathway plays an important role in ROS homeostasis. In *Arabidopsis*, overexpression of MKK1 enhances the activity of the MAPK cascade, which is also activated by ROS [14], [15]. Xing *et al*. found that overexpression of MKK1 decreases stress-associated ROS levels and increases salt and drought tolerance, conversely, MKK1 deficiency results in elevated ROS production as well as increased stress sensitivity [16]. As previously described, AtMKK1 and AtMKK4 have both been implicates in ROS signaling [17]. Additionally, AtMKK3, a calmodulin-dependent activator of MPK8, participates in ROS homeostasis [18]. How MAPKKs participate in ROS signaling is a question that needs to be further pursued in the future.

Cotton (*Gossypium hirsutum*), which an important crop around the world, has been cultivated for its fiber. It also serves as a significant source of feed, foodstuffs, oil, and biofuel. Various abiotic and biotic factors have been shown to inhibit the growth and yield of this species [12], [19]. MAPKKs can be divided into at least four groups, designated A–D. Group A MAPKKs may be involved in various pathways and exhibit multiple biological functions [20]. Although the roles of MAPKKs in the response to environmental stresses have been well studied in plants, functional information on the MAPKKs present in cotton is scarce. Given that MAPK cascades are prone to cross-talk, we exploited molecular and genetic approaches to study a novel group A MAPKK gene from cotton, *GhMKK1*, to gain a deeper understanding of the functions of the MAPKKs in cotton. We report that *GhMKK1* is a crucial regulator of the response to environmental stresses, and *GhMKK1* expression markedly enhances tolerance to salt and drought stresses in *N. benthamiana*. In addition, *GhMKK1*-overexpression in plants can either inhibit ROS production or lead to efficient scavenges of the excess ROS produced due to external stresses. Overexpression of *GhMKK1* also elevates the susceptibility of plants to pathogens. All of these results indicate that *GhMKK1* plays a functional role in multiple mechanisms through which plants respond to abiotic and biotic stresses. These findings further broaden our knowledge of the role of *GhMKK1* in signal transduction.

## Experimental Procedures

### Biological materials, growth conditions, and treatments

Cotton (*Gossypium hirsutum* L. cv. lumian 22) seeds were placed in wet carbasus to accelerate germination, and the seedlings were then transplanted to controlled environmental conditions and grown at 25±1°C with a 16 h light/8 h dark cycle (relative humidity of 60–75% and fluorescent light intensity of ∼200 µmol m^−2^ per second). Additionally, *Nicotiana benthamiana* seeds were surface-sterilized and germinated on Murashige-Skoog (MS) medium under greenhouse conditions. Two- or three-leaf stage *N. benthamiana* seedlings were then transplanted into soil and further maintained under greenhouse conditions. For the various treatments described hereafter, seven-day old cotton seedlings were used. NaCl, wounding, H_2_O_2_, and salicylic acid (SA) treatments were performed as described previously [21]. For temperature treatments, uniformly developed cotton seedlings were transferred to 4°C for given time periods. For the other treatments, developed seedlings were sprayed with solutions containing the indicated concentrations of polyethylene glycol (PEG), abscisic acid (ABA), salicylic acid (SA), H_2_O_2_, methyl jasmonate (MeJA), ethephon (ET), or *Ralstonia solanacearum* for the given time periods. The treated cotyledons were collected for RNA extraction, and the roots, stems, and leaves were harvested at the appropriate time points and frozen in liquid nitrogen. Each treatment was repeated at least twice.

### *GhMKK1* cloning, vector construction, and plant transformation

Under the control of the *Cauliflower mosaic virus* (CaMV) 35S promoter, the *GhMKK1* cDNA sequence (GenBank accession number: HQ828075) was inserted into the binary pBI121 vector via the *Xba*I and *Sal*I sites. The recombinant plasmid was then introduced into *Agrobacterium tumefaciens* (strain LBA4404), and transformantion of *N. benthamiana* was performed using the leaf disc method [22]. The transgenic seedlings were selected on MS agar medium containing 100 mg/L of kanamycin, then transferred to soil and grown in a greenhouse. The seeds of the transgenic lines were harvested from inbred lines. The transgenic T_3_ lines were used in the experiments, and plants transformed with the empty pBI121 vector were employed as controls. All of the primers used in this study are listed in Table 1.

**Table 1:** Oligonucleotide primers used in this study.

Primer	Primer sequence (5′→3′)
The cloning of the full-length cDNA	
Internal degenerate primers	(D = A, G, or T; N = A, C, G, or T; R = A or G; V = A, C, or G; Y = C or T)
M1	GGVACDTTYAARGATGGDGATC
M2	CCCAARCTCCADATRTCRC
5′-RACE primers	
5P1	CTGATGGCTTAATGGGCGG
5P2	GCACAATTCCACCATTACCC
5P3	GGACACTGAGAGGACTGG
AAP	GGCCACGCGTCGACTAGTACG(G)_14_
AUAP	GGCCACGCGTCGACTAGTACG
3′-RACE primers	
3P1	CCTCAGGGCTAGCAAACAC
3P2	CCCGAACCCTATCTTGCTG
3P3	CCAGTCCTCTCAGTGTCC
B26	GACTCGAGTCGACATCGA(T)_18_
B25	GACTCGAGTCGACATCGA
The full-length cDNA primers	
Q1	GAAGAAGAAGCAAAACCTCAGATG
Q2	GTCATCACTACAGCCGCTC
The cloning of the genomic sequence	
Q1	GAAGAAGAAGCAAAACCTCAGATG
Q2	GTCATCACTACAGCCGCTC
Primers used in expressin vector	
Z1	TCTAGAGATGAAGAAGGGAAAGGG
Z2	GTCGACGTCATCACTACAGC
35SF	CCAAGAAGGTTAAAGATGCAG
35SR	GAAGACGTGGTTTTAACG
*β*-actin F	TGGACTCTGGTGATGGTGTC
*β*-actin R	CCTCCAATCCAAACACTGTA

### RNA extraction and semi-quantitative RT-PCR analyses

Total RNA was extracted from the cotton seedlings and *N. benthamiana* leaves using the CTAB method and the TRIzol Reagent (Invitrogen, Carlsbad, CA, USA), respectively. The CTAB extraction buffer and the protocol were modified from Wang and Stegemann [23]. Samples from cotton seedlings were ground into a fine powder, then transferred to tubes filled with 650 µL of pre-warmed CTAB extraction buffer and vortexed for 1 min at 25°C. An equal volume of phenol-chloroform-isoamyl alcohol (25∶24∶1) was next added to the tubes, which were then mixed well and centrifuged for 15 min at 12,500 rpm at 4°C. After centrifugation, the supernatant was mixed with LiCl and stored at 4°C overnight. The next day, the samples were centrifuged, and the obtained pellet was dissolved in 50 µL of RNase-free water. Subsequently, 5 µL of DNase I Buffer, 2 µL of DNase I, and 1 µL of PRI (TaKaRa, Dalian, China) were added to digest the DNA at 37°C for 30 min. RNase-free water (150 µL) and an equal volume of phenol-chloroform-isoamyl alcohol were added, followed by centrifugation. NaAc and ethanol were then added to the supernatants, and they were held at −80°C for 1 h. After washing with 75% ethanol, the pellets were dissolved in 50 µL of RNase-free water, and the obtained RNA was stored at −80°C. Total RNA was extracted from *N. benthamiana* leaves using the TRIzol Reagent according to the manufacturer's instructions. This RNA was employed for first-strand cDNA synthesis using a reverse transcriptase system (TransGen Biotech, Beijing, China). Amplifications were performed using the following thermocyling program: 94°C for 5 min, followed by 26–32 cycles of 94°C for 40 s, 50–55°C for 40 s, and 72°C for 40 s. The obtained PCR products were separated in a 1.8% agarose gel and visualized via ethidium bromide staining. The *18S rRNA* gene (GenBank accession number: U42827.1) or the *N. benthamiana β-actin* gene (GenBank accession number: JQ256516.1) was used as a loading control in each reaction. All gene information in RT-PCR are listed in Table S1 and Table S2.

### Subcellular localization of the 35S-GhMKK1::GFP fusion protein

The open reading frame (ORF) of *GhMKK1* was amplified using the specific primers Q1/Q2. The resultant fragment was inserted into the binary vector pBI121-GFP, which fused the C-terminaus of the *GhMKK1* gene with the N-terminus of the green fluorescence protein (GFP) gene; this fusion protein was controlled by the Cauliflower mosaic virus (CaMV) 35S promoter. For transient expression, the plasmid DNA was transformed into onion inner epidermal cells using a previously described particle bombardment method [24]. After particle bombardment, the tissues were incubated on MS agar medium under dark conditions at 25°C for 10–12 h, and the 35S-GFP plasmid was used as a control. Each construct was analyzed at least three times. The obtained fluorescence pattern was observed using a confocal laser scanning microscope (LSM 510 META, ZEISS, Germany).

### 3,3′-Diaminobenzidine (DAB), Nitro Blue tetrazolium (NBT), and trypan blue staining assays

For DAB staining, the *N. benthamiana* leaves were incubated in DAB solution (1 mg/mL, pH 3.8) for 24 h at 25°C in the dark. After staining, the leaves were soaked in 95% ethanol overnight to remove chlorophyll [25]. Superoxide anion radicals were detected via NBT staining, according to Jabs *et al*. [26]. In the NBT assays, collected leaves were incubated in NBT solution (0.1 mg/mL) for 24 h at 25°C in the dark. After staining, the leaves were soaked in 95% ethanol overnight to remove chlorophyll. Seedlings treated with water were used as controls. For trypan blue staining, leaves were removed after treatment and stained with lactophenol-trypan blue (10 mL of lactic acid, 10 mL of glycerol, 10 g of phenol, and 10 mg of trypan blue, all dissolved in 10 mL of distilled water), then mixed with ethanol in a 1∶1 proportion (v/v). Whole leaves were boiled for approximately 5 min in the staining solution and soaked in chloral hydrate (2.5 g of chloral hydrate dissolved in 1 mL of distilled water) for 12 h to remove chlorophyll [27].

### Oxidative stress experiments

For oxidative damage tests, leaf disks 1.3 cm in diameter were excised from healthy, fully expanded leaves from 2-month-old transgenic plants. The disks were floated in 10 mL of solutions with different concentrations of Methyl Viologen (MV) (0, 200, and 400 µM) for 24 h, the disks were then immersed in 95% ethanol for 24 h to extract chlorophyll. Chlorophyll a and b contents were determined through spectrophotometric measurements. Determination of O_2_^−^ production in the leaves of both treated and control transgenic *N. benthamiana* plants was performed according to the method described by Xing *et al*. [28]. Malondialdehyde (MDA) concentrations were used as a biomarker for oxidative stress. The measurements were carried out as described previously [29]. Proline accumulation following drought stress was monitored at the indicated times, as described by Shan *et al*. [30]. These experiments were repeated at least three times.

### Determination of total protein contents and antioxidant enzyme activities

After 8 days without water, 0.5 g samples of the transgenic seedling leaves were collected to perform CAT, APX, POD, and SOD measurements, using the method described by Leclercq *et al*. [31]. Total protein concentrations were determined according to the Bradford method [32].

### Pathogen infection

The bacterial pathogen *Ralstonia solanacearum* (*R. solanacearum*) was cultured overnight at 37°C in Luria–Bertani (LB) broth, then harvested via centrifugation, and resuspended in sterile tap water. The fungal pathogen *Rhizoctonia solani* (*R. solani*) was cultured on potato dextrose agar (PDA) medium at 28°C for 2 weeks, and the spores were then suspended in 1% glucose. To perform the disease resistance analysis, an *R. solanacearum* bacterial suspension (OD_600_ = 0.6–0.8) or *R. solani* spore suspension (10^5^ spores/mL) was infiltrated into leaves detached from 8-week-old T_3_ *GhMKK1*-overexpressing (OE) or wild-type (WT) plants using a needleless syringe. At least three independent experiments were performed for all pathogen inoculations.

## Results

### Isolation and sequence characterization of *GhMKK1*

Based on the conserved kinase subdomain of the known MKKs, a pair of degenerate primers (M1/M2) was designed to isolate a cDNA fragment from cotton cotyledons. Then, three sets of specific primers, 5P1/5P2/5P3, 3P1/3P2/3P3, and Q1/Q2, were used for 5′ rapid amplification of cDNA ends (RACE), 3′ RACE-PCR, and identification of the full-length cDNA sequence, respectively. The deduced full-length sequence of the MKK cDNA fragment consisted of 1,234 nucleotides, including a 21 bp 5′-untranslated region (UTR), a 127 bp 3′-UTR, and 1,086 bp open reading frame (ORF). This ORF encodes a polypeptide of 361 amino acid with a calculated molecular mass of 39.945 kDa and an isoelectric point of 5.08. According to the nomenclature for plant MAPKs [1] and because this clone exhibited a high level of sequence similarity to *Arabidopsis* AtMKK1, we designated this gene *GhMKK1* (GenBank accession number: HQ828075).

**Figure 1:**
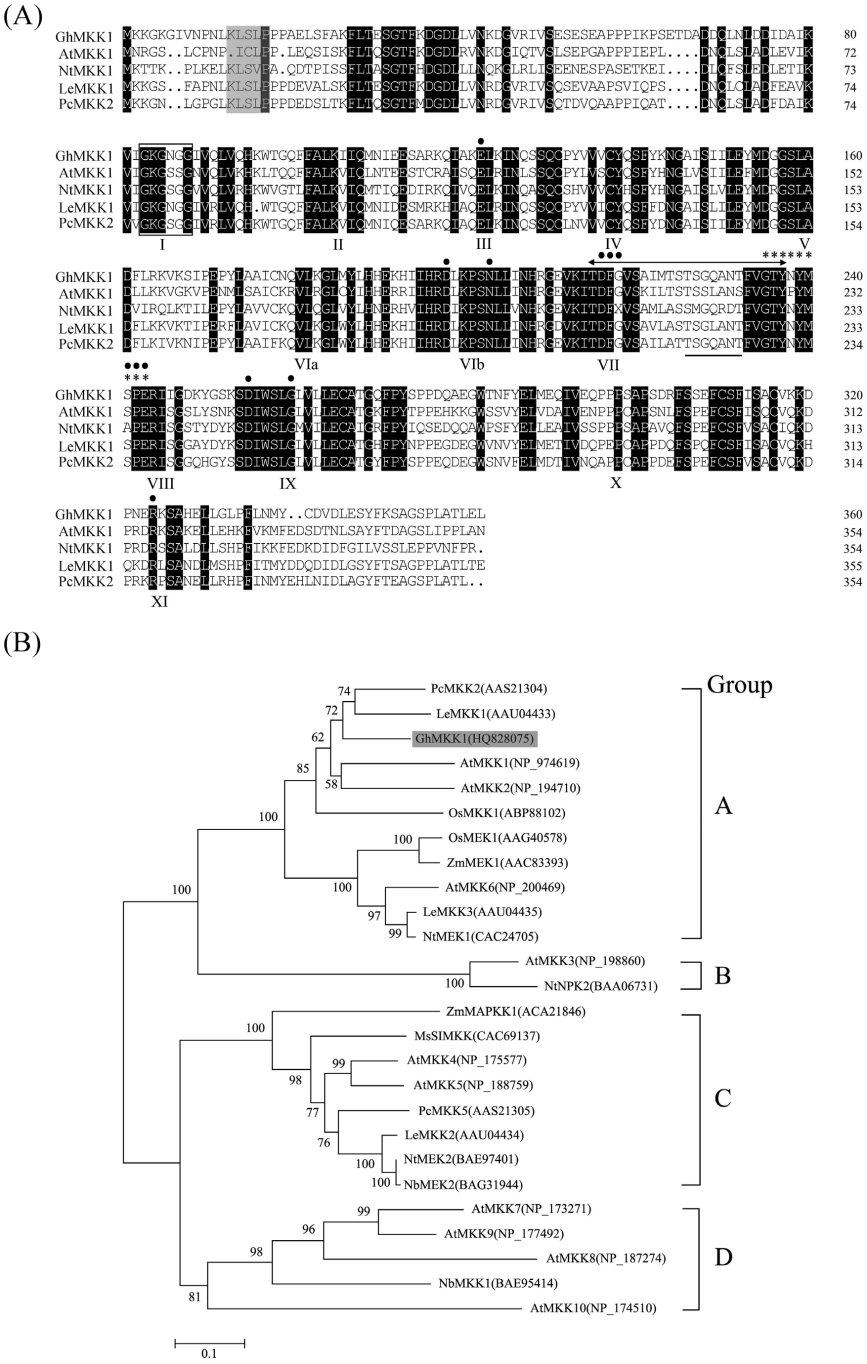
Characterization and sequence analysis of GhMKK1. (A) Multiple amino acid sequence alignment of GhMKK1 (HQ828075) with AtMKK1 (NP_974619), NtMEK1 (CAC24705), LeMKK1 (AAU04433), and PcMKK2 (AAS21304). Identical and similar amino acids are shaded in black, and the docking domain is shaded in grey. The Roman numbers (I-XI) on the bottom indicate conserved subdomains, and a double-head arrow marks the active site motif. The serine and/or threonine residues in the conserved consensus motif, S/TXXXXXS/T, between subdomains VII and VIII of the MAPKKs are underlined, and the conserved consensus motif GXGXXG is boxed. The dots above the sequences represent activating sites, and asterisks indicate substrate specificity. (B) The phylogenetic relationships between GhMKK1 and other plant MAPKK proteins. The neighbor-joining phylogenetic tree was created using Clustal W and MEGA 4.0 software. The numbers above or below the branches indicate the bootstrap values (>50%) from 500 replicates. GhMKK1 is highlighted in grey. Each gene name is followed by its protein ID. The species of origin is indicated by the abbreviation before the gene names: Pc, *Petroselinum crispum*; Le, *Lycopersicon esculentum*; Gh, *Gossypium hirsutum*; At, *Arabidopsis thaliana*; Os, *Oryza sativa*; Zm, *Zea mays*; Nt, *Nicotiana tabacum*; Ms, *Medicago sativa*; and Nb, *Nicotiana benthamiana*. A, B, C, and D indicate the MAPKK group.

Multiple protein sequence alignment against MAPKKs from various plants showed that GhMKK1 shared high homology with AtMKK1. Comparision with other plant MAPKKs showed that the GhMKK1 protein possessed fully canonical motif structures (Fig. 1A), including 11 conserved sub-domains that are important for its catalytic function, the consensus sequence of S/TXXXXXS/T between the VII and VIII domains, a catalytic loop (activation loop), and a docking domain (D-domain). D-domains (also referred to as D box or DEJL motifs) bind MAPKs or phosphatases [33].

Plant MKK proteins are classified into four groups, designated A–D [20]. To further investigate the evolutionary relationship of the cloned MKK with the other known plant MKKs, a phylogenetic analysis was performed with the obtained amino acid sequences using the Neighbor-Joining method with MEGA version 4.0 software. The analysis showed that GhMKK1 exhibited a high similarity to members of the group A MKKs, such as PcMKK2, LeMKK1, AtMKK1, and AtMKK2 (Fig. 1B).

To investigate the genomic structure of *GhMKK1*, a 2,720 bp genomic fragment of *GhMKK1* (GenBank accession number: JX534501) was isolated from cotton genomic DNA using the specific primers Q1/Q2. Sequence comparison revealed that *GhMKK1* displayed seven introns, similar to the group A MKKs (Table 2). The obtained phylogenetic tree further indicated that GhMKK1 was a member of the MKKs, clustered within group A.

**Table 2:** The lengths of the exons and introns in *GhMKK1* and *AtMKK1*.

Gene	Exon	Length (bp)	Intron	Length
*GhMKK1*	1	83	1	100
	2	85	2	182
	3	147	3	578
	4	225	4	83
	5	183	5	123
	6	224	6	264
	7	138	7	156
	8	1		
*AtMKK1*	1	71	1	109
	2	85	2	279
	3	138	3	86
	4	224	4	86
	5	181	5	99
	6	224	6	108
	7	47	7	83
	8	95	8	93

### Subcellular localization of *GhMKK1*

**Figure 2:**
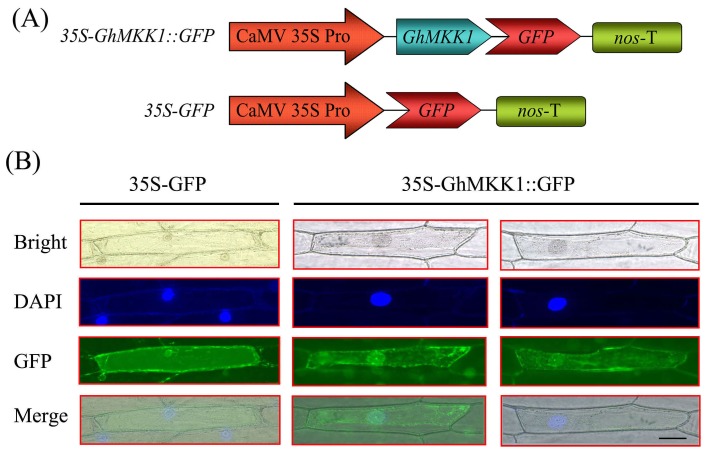
Subcellular localization of the GhMKK1 protein transiently expressed in onion epidermal cells. (A) Schematic diagram of the 35S-GhMKK1::GFP fusion construct and the control 35S-GFP construct. (B) Transient expression of the 35S-GhMKK1::GFP and 35S-GFP constructs in onion epidermal cells. Green fluorescence was observed using a confocal microscope 12 h after particle bombardment. The nuclei of the onion cells were visualized via DAPI staining. Bar  = 200 µm.

In silico analysis using the Plant-mPLoc program predicted that the GhMKK1 protein would localize to the nucleus, whereas, the PSORT program predicted that GhMKK1 would localize to the cytoplasm. To determine which of these predictions was correct, the 35S-GhMKK1::GFP fusion protein and a 35S-GFP fusion protein (as a control) were constructed to investigate the subcellular localization of GhMKK1 (Fig. 2A). These two constructs were individually introduced into onion epidermal cells through particle bombardment. The fluorescence of both 35S-GhMKK1::GFP and 35S-GFP was detected in both the nucleus and the cytoplasm (Fig. 2B). These results revealed that the GhMKK1 protein is found in the nucleus and in the cytoplasm, which might provide a structural basis for the proposed function of the protein.

### Differential expression patterns of *GhMKK1* in different organs and under different stresses

**Figure 3:**
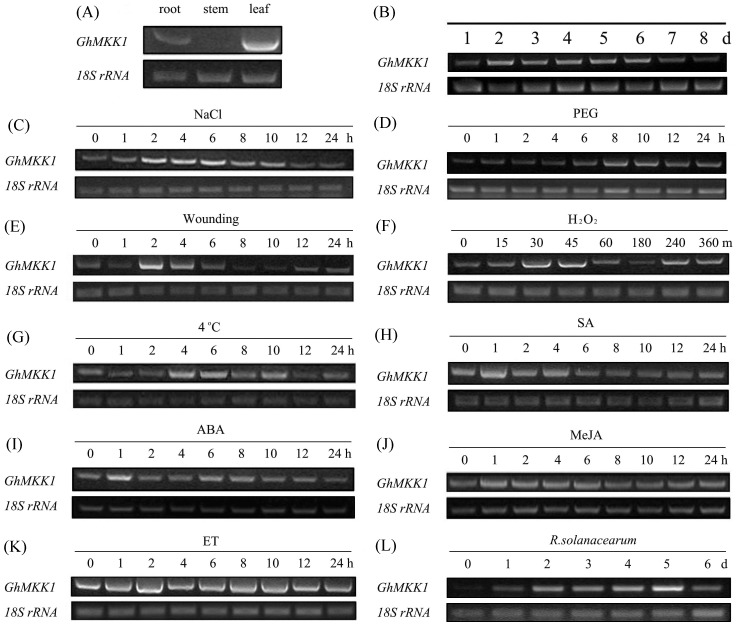
Expression patterns of *GhMKK1* in different tissues, developmental stages and under different stress conditions. (A) The tissue-specific expression of *GhMKK1* was analysed via RT-PCR using total RNA extracted from the roots, stems, and cotyledon leaves of 7-day-old cotton seedlings. (B) The expression profiles of *GhMKK1* were measured in the cotyledon leaves of 7-day-old cotton seedlings. For the stress treatments, 7-day-old cotton seedlings were obtained from a hydroponic culture and were subjected to treatment with 100 mM NaCl (C), 15% PEG (D), wounding (E), 100 µM H_2_O_2_ (F), low temperature (4°C) (G), 2 mM SA (H), 100 µM ABA (I), 100 µM MeJA (J), 100 µM ET (K), release from ethephon, and *R. solanacearum* infection (L). Total RNA was isolated at the indicated times following the initiation of treatments and was subjected to RT-PCR analysis. The obtained PCR products were visualized via agarose gel electrophoresis, followed by ethidium bromide staining. The *18S rRNA* gene was employed as an internal control. This experiment was repeated at least twice.

To identify the organ-specific expression patterns of *GhMKK1*, RT-PCR was carried out using 7-day-old cotton seedlings obtained from hydroponic cultures. As shown in Fig. 3A, the majority of *GhMKK1* expression was found in the cotyledon leaves, followed by the roots and stems, which showed that *GhMKK1* displayed an organ-specific expression pattern. At the same time, the expression of *GhMKK1* in the seedling stage was found to be negligible (Fig. 3B).

To determine the expression pattern of *GhMKK1* following various environmental stresses, the cotton seedlings were exposed to abiotic stresses such as NaCl, PEG, wounding, H_2_O_2_, and 4°C chilling treatments. As shown in Fig. 3C–G, the NaCl, wounding, and H_2_O_2_ treatments significantly induced the expression of *GhMKK1*, and the NaCl treatment resulted in a relatively persistent effect from 2 to 10 h. The expression of *GhMKK1* was rapidly and transiently enhanced after the seedlings were wounded, and there was no change in *GhMKK1* transcript levels detected during the 6 to 24 h series. Following PEG and 4°C treatment, there was a mild accumulation of *GhMKK1* transcripts. In addition, we measured the expression of *GhMKK1* in cotton seedlings under biotic stresses, such as infection with *R. solanacearu*. This type of stress led to a significant induction of *GhMKK1* on day 2, which peaked on day 5 of the time course (Fig. 3L). These data suggest that *GhMKK1* transcript levels are responsive to environmental stresses and might play pivotal roles in the plant stress response.

SA, ABA, MeJA, and ET, which are involved in diverse signaling pathways, were also examined to explore the *GhMKK1* signal transduction mechanism that underlies the response to environmental stresses (Fig. 3H–K). The transcription of *GhMKK1* peaked 1 h after ABA treatment, then declined back to its initial level. There was a slight negative effect on the accumulation of *GhMKK1* observed upon SA treatment. In addition, MeJA and ET had no clear effect on the transcription of *GhMKK1*. Taken together, these results indicate that *GhMKK1* may be responsible for mediating defence-related signal molecules and may play a role in multiple plant defence transduction pathways.

### Overexpression of *GhMKK1* increases the salt tolerance of transgenic plants

**Figure 4:**
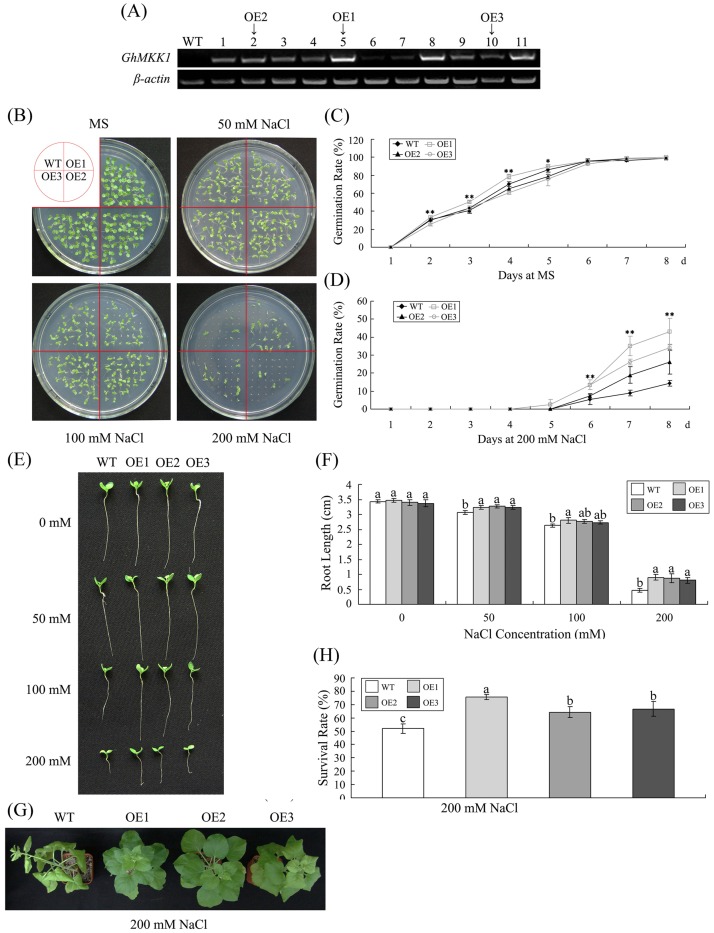
Salt tolerance test comparing the wild-type and *GhMKK1*-overexpressing *N. benthamiana* plants. (A) Analysis of *GhMKK1* expression in wild-type (WT) and T_1_ OE plants. (B) Seed germination on MS medium containing different concentrations of NaCl. (C–D) Germination rates of the WT and OE lines under normal and NaCl treatment conditions. Based on daily scoring, the results obtained using MS medium containing 200 mM NaCl are presented. The presented data are the means ±SE of three independent experiments (*n* = 3). Asterisks (* or **) above the lines indicate significant differences (*P<0.05; **P<0.01) according to Duncan's multiple range test performed in SAS version 9.1 software. (E) Post-germination seedling development of the WT and the OE lines on MS supplemented with different concentrations of NaCl. The seeds sown on MS medium that showed radicle emergence after 3 d were transferred to MS medium containing different concentrations of NaCl. The plates were oriented vertically, with seedlings kept upside down, and a photograph was taken 14 d after transfer. (F) Primary root lengths of the seedlings 14 d after germination in the presence of different NaCl concentrations. The presented data are the means ±SE of three independent experiments (*n* = 6). Different letters above the columns indicate significant differences (*P*<0.05) according to Duncan's multiple range test performed using SAS version 9.1 software. (G) Photograph of representative 10-week-old WT and OE plants grown in soil containing 200 mM NaCl for 14 d. (H) Survival rates of 10-week-old plants treated with 200 mM NaCl for 14 d. The presented data are the means ±SE of three independent experiments (*n* = 6). Different letters above the columns indicate significant differences (*P*<0.01) according to Duncan's multiple range test performed using SAS version 9.1 software.

To investigate the function of *GhMKK1* in response to environmental stresses, *GhMKK1* was overexpressed in transgenic *N. benthamiana* plants. Through kanamycin resistance selection, a total of 20 independent transgenic lines were obtained; these lines were confirmed using PCR. Subsequently, eleven lines of transgenic T1 plants were selected to detect the levels of *GhMKK1* expression in leaf tissue (Fig. 4A). Compared to the control, no visible alternation in morphology was observed in the wild-type (WT) plants (data not shown). However, three representative lines overexpressing *GhMKK1*, OE1 (5#), OE2 (2#), and OE3 (10#) exhibited different levels of *GhMKK1* expression. The T3 progeny of these transgenic plants were randomly selected for further investigation.

To explore whether constitutive expression of *GhMKK1* in transgenic plants effects their salt tolerance, we first studied the transgenic plants' salt tolerance during the germination and seedling stages of development. Seeds from the wild-type plants and the three homozygous transgenic lines (OE1, OE2, and OE3) were surface sterilized and germinated on MS agar medium containing 0, 50, 100, or 200 mM NaCl. As shown in Fig. 4B and 4C, there was no visible difference between the wild-type and transgenic lines following treatment with 0, 50 and 100 mM NaCl. When the NaCl concentration was increased to 200 mM, ∼14% of the wide-type seeds and ∼26–43% of the transgenic seeds germinated after 8 days (Fig. 4D). To further confirm the transgenic phenotypes, the seeds of the wild-type and the transgenic plants were germinated on MS agar medium for 3 days, and were then transferred to medium supplemented with various NaCl concentrations (0, 50, 100, or 200 mM). The data indicated the root length of each plant under the established conditions. There was no significant difference between the wild-type and transgenic lines treated when they were with 0 or 50 mM NaCl. However, the root growth was much greater in the transgenic lines than in the wild-type plants when grown on 100 or 200 mM NaCl (Fig. 4E and 4F).

To verify that *GhMKK1* enhanced the salt tolerance of the transgenic plants, the wild-type and T_3_ transgenic plants were irrigated with salt water (200 mM NaCl) and grown in soil in a greenhouse. After two weeks, severe growth retardation was observed in the wild-type plants, whereas the inhibition of growth was relatively less evident in the transgenic plants exposed to 200 mM NaCl. The leaves of the wild-type plants became wilted and curled (Fig. 4G), and we found that only ∼50% of the wild-type plants survived, and the survival rate of the wild-type plants was less than that of the transgenic plants, with almost all of the transgenic plants surviving (Fig. 4H). These data show that expression of *GhMKK1* alleviated the positive effects imposed by salt stress in the transgenic plants during both seed germination and vegetative growth.

### *GhMKK1*-overexpressing plants display increased drought tolerance

**Figure 5:**
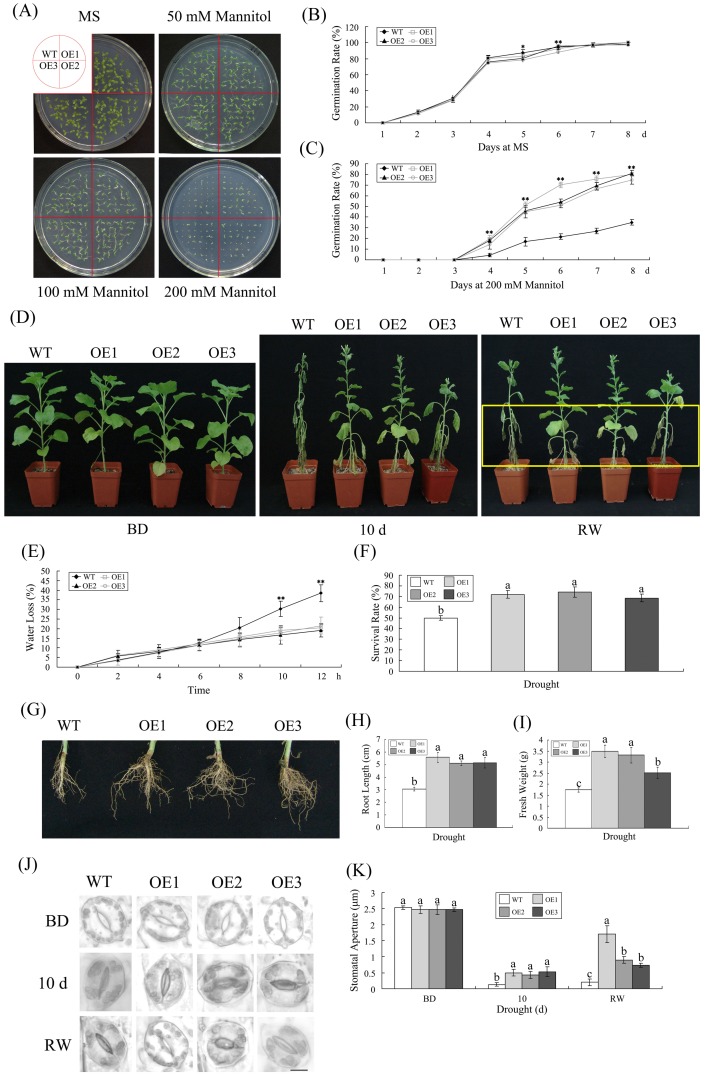
Drought tolerance test comparing the wild-type and the *GhMKK1*-overexpressing *N. benthamiana* plants. (A) Seed germination on MS medium with 0, 50, 100, or 200 mM mannitol. (B–C) Germination rates of the WT and OE lines under normal and mannitol treatment conditions. Based on daily scoring, the results obtained on the MS medium containing 200 mM mannitol are presented. The presented data are the means ±SE of three independent experiments (*n* = 3). Asterisks (* or **) above the lines indicate (highly) significant differences (*P<0.05; **P<0.01) according to Duncan's multiple range test performed using SAS version 9.1 software. (D) Photograph of representative 10-week-old WT and OE plants grown in soil under drought conditions for 10 d, then watered for 2 d to allow them to recover. BD, before drought treatment; RW, rewatering. (E) The water loss from the detached leaves of WT and OE plants at the indicated times. The rate of water loss was calculated by the loss of fresh weight in the samples. The presented data are the means ±SE of three independent experiments (*n* = 6). Asterisks (**) above the lines indicate (highly) significant differences (*P*<0.01) according to Duncan's multiple range test performed using SAS version 9.1 software. (F) Survival rates of WT and OE plants under drought stress. The presented data are the means ±SE of three independent experiments (*n*≥50). Different letters above the columns indicate significant differences (*P*<0.0001) according to Duncan's multiple range test performed using SAS version 9.1 software. (G–I) Phenotype of roots subjected to drought stress for WT and OE plants, together with additional root lengths and fresh weights. The presented data are the means ±SE of three independent experiments (*n* = 6). Different letters above the columns indicate significant differences (*P*<0.0001) according to Duncan's multiple range test performed using SAS version 9.1 software. (J–K) Stomatal changes observed with a microscope before and after drought treatment. The stomatal aperture is displayed. BD, before drought treatment; RW, rewatering. Bar  = 200 µm. The presented data are the means ±SE of three independent experiments (*n* = 6). Different letters above the columns indicate significant differences (*P*<0.01) according to Duncan's multiple range test performed using SAS version 9.1 software.

Because salt stress results in osmotic stress in plants cells, we performed drought tolerance tests in both the wild-type and transgenic plants to gain a better understanding of *GhMKK1* under drought conditions. First, seeds were germinated to capacity on MS agar medium containing different concentrations of exogenous mannitol (0, 50, 100, or 200 mM) to mimic drought stress. No obvious morphological differences were visible in the seedlings when they were treated with 0 to 100 mM NaCl. However, following treatment with 200 mM mannitol, the germination of both the wild-type and transgenic lines was inhibited, and wild-type seeds were more strongly suppressed than transgenic seeds, as only ∼35% of the wild-type seeds germinated compared to ∼80% of the transgenic seeds 8 days after sowing (Fig. 5A–C). Next, wild-type and transgenic plants were examined to confirm their drought tolerance at the vegetable growth stage. The wild-type and T_3_ transgenic plants were grown in the same pot without water for 10 days. After 10 days of drought treatment, the wild-type plants were completely wilted, whrease the transgenic plants were less affected (Fig. 5D). The transgenic plants recovered more rapidly than the wild-type plants when they were watered for 2 days following the drought treatment, though several of their leaves did not recover completely. Additionally, the rate of water loss from the detached leaves of the wild-type plants was ∼38%, which was nearly twice the amount of water loss observed in the transgenic plants (∼20%). Furthermore, the survival rate of the transgenic plants was ∼20% higher than that of the wild-type plants (Fig. 5E and 5F).

Plants control water loss through well-developed root systems and by regulating stomatal closure during drought stress. As shown in Fig. 5G–I, the transgenic plants exhibited longer and thicker roots than the wild-type plants as well as a higher fresh weight. However, there was no significant difference observed in the stomatal aperture between the transgenic and the wild-type lines under normal conditions, neverthless, almost all of the stomata of the wild-type plants closed after drought stress, and the stomatal apertures in the transgenic lines were more open than those of the wild-type plants (Fig. 5J and 5K). After 2 days of watering and recovery, the stomatal apertures of the transgenic plants were more than 5 times wider, on average, than those of the wild-type plants. In summary, these results suggest that overexpression of *GhMKK1* can enhance the drought tolerance of the transgenic plants at both the seedling and the vegetable growth stages.

### *GhMKK1* mediates the accumulation of ROS in transgenic plants

**Figure 6:**
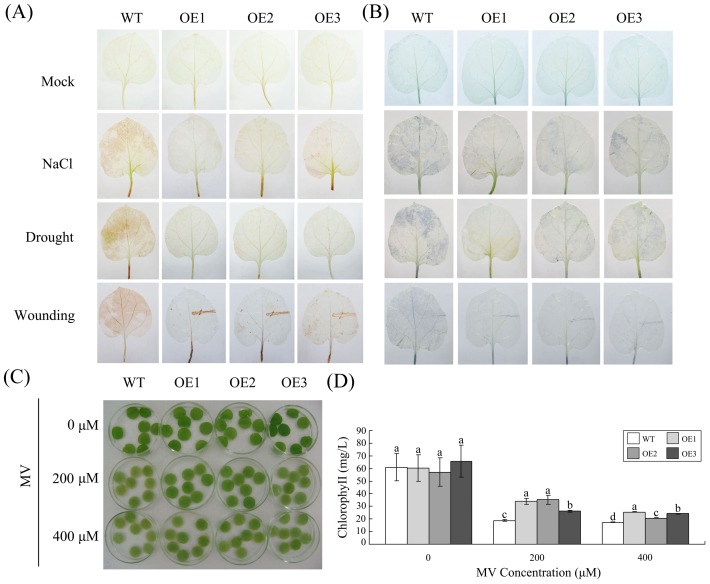
Analysis of ROS accumulation in WT and OE plants in response to abiotic stresses. (A–B) Abiotic stress-induced H_2_O_2_ and O_2_^−^ accumulation detected via DAB staining and NBT staining, respectively. (C) Leaf disks from WT and OE plants were incubated in different concentrations of MV (0, 200, or 400 µM) under greenhouse conditions. (D) Relative chlorophyll contents were determined in the leaf disks of WT and OE plants following MV treatments. Disks floated in water were used as a control. The presented data are the means ±SE of three independent experiments (*n* = 6). Different letters above the columns indicate significant differences (*P*<0.0001) according to Duncan's multiple range test performed using SAS version 9.1 software.

Cellular ROS can be induced by many types of environmental stresses, such as salt and drought, among other types of stress [34], [35]. To investigate whether *GhMKK1* expression conferred an elevated tolerance to oxidative stress in the transgenic plants, detached leaves from the wild-type and the transgenic plants subjected to NaCl, drought, and wounding stresses were used to measure the accumulation of H_2_O_2_ and O_2_^−^ using the 3,3′-diaminobenzidine (DAB) and Nitro Blue tetrazolium (NBT) staining methods, respectively. Leaves detached from untreated wild-type and the transgenic plants were used as mock controls. Under normal growth conditions (Fig. 6A and 6B), the H_2_O_2_ contents of the wild-type and the transgenic plants were similar. However, following treatment with NaCl, drought and wounding, the DAB staining was found to be much lighter in the transgenic plants, indicating that the level of H_2_O_2_ was considerably lower in the transgenic plants compared to the mock-treated plants. Similar to what was observed for DAB staining, NBT staining revealed that there was less O_2_^−^ accumulation in the leaves from the transgenic plants.

To illustrate how *GhMKK1* overexpression leads to oxidative resistance, leaf disks from the wild-type and the transgenic plants were exposed to increasing concentrations of MV (0, 200, or 400 µM) for 48 h to stimulate oxidative stress. The results presented in Fig. 6C and 6D show that the transgenic plants exhibited intense oxidative resistance. Leaf disks from the wild-type plants changed from green to almost yellow in color following oxidative stress treatment, while the disks from the transgenic plants displayed oxidative tolerance to the MV-induced damage. All of these data indicate that the transgenic plants produce lower levels of ROS, suggesting that overexpression of *GhMKK1* either inhibits ROS production or results in the efficient scavenging of excess ROS.

### *GhMKK1* overexpression enhances oxidative stress tolerance in plants

To ascerain the possible mechanisms underlying the enhanced antioxidant defence ability observed in the transgenic plants, we examined several physiological indexes and measured the enzymatic activity of genes that encoded ROS-scavenging enzymes in both the transgenic plants and the control plants under drought stress.

Excess ROS produces superoxide radicals, including superoxide anions (O_2_^−^), which are harmful to plants. Lipid hydroperoxidation contributes to and serves as an indicator of cellular oxidative damage [36]. Proline accumulation in response to osmotic stress has also been well documented in both prokaryotic and eukaryotic organisms. By acting as a hydroxyl radical scavenger, proline contributes to osmotic adjustment and protects macromolecules during dehydration [29]. As shown in Table 3, while no remarkable difference was observable between the untreated wide-type and the untreated transgenic plants, the O_2_^−^ and MDA contents in the wide-type plants were dramatically increased compared to the transgenic plants under drought stress. However, there was little proline detected in any of the untreated plant lines. While the proline concentration dramatically increased with drought stress, there was a discrepancy in the results obtained between the transgenic lines.

**Table 3:** Several physiological indexes in WT and OE plants under drought stress.

		WT	OE1	OE2	OE3
O_2_^−^(nmol/g FW)	No treatment	247.143±21.666	149.038±3.364	200.666±9.664	212.373±14.225
	drought	848.929±23.690	479.591±13.591	569.981±14.069	697.965±14.075
MDA (µmol/g FW)	No treatment	1.727±0.015	1.936±0.022	1.921±0.079	1.457±0.075
	drought	6.598±0.503	2.492±0.347	3.058±0.267	3.957±0.219
Proline (µg/g FW)	No treatment	71.332±14.736	64.810±10.171	55.219±5.857	43.569±8.959
	drought	548.860±10.386	721.036±36.276	645.841±29.351	775.031±7.453
CAT (U/g FW)	No treatment	58.104±2.236	41.830±3.947	62.409±1.794	51.751±15.923
	drought	59.163±6.120	109.491±0.692	76.576±3.078	89.929±17.494
APX (U/g FW)	No treatment	115.562±0.645	92.032±1.739	120.749±2.713	86.125±1.883
	drought	98.606±1.360	139.470±3.539	111.506±5.498	122.658±3.175
POD (U/g FW)	No treatment	162.225±2.332	157.520±0.733	167.722±1.106	164.832±1.141
	drought	144.315±10.475	238.443±2.906	189.655±2.235	163.729±3.568
SOD (U/g FW)	No treatment	85.413±5.885	110.790±6.223	105.345±12.074	135.244±8.130
	drought	145.426±6.863	108.173±9.321	113.548±10.780	122.367±7.507

To determine the nature of the antioxidant response to drought stress caused by *GhMKK1* overexpression, the enzymatic activities of catalase (CAT), ascorbate peroxidase (APX), peroxidase (POD) and superoxide dismutase (SOD) were monitored under both normal and drought conditions (Table 3). Prior to drought stress, there were no notable differences in POD activity between any of the lines. However, the transgenic lines displayed a significant increase in POD activity when maintained under drought conditions. Likewise, the activities of CAT and SOD were greatly increased in the transgenic lines compared to the wild-type plants under drought stress. Drought treatment also markedly increased APX activity in the transgenic plants, with the exception of the OE2 line, which displayed a higher APX activity than the wild-type plants. Briefly, these results indicate that *GhMKK1*-overexpression enhanced the antioxidant ability of multiple antioxidant enzymes in the transgenic plants, suggesting that *GhMKK1* might play a role in the regulation of the ROS network pathway.

### *GhMKK1*-overexpressing plants exhibit enhanced pathogen susceptibility

**Figure 7:**
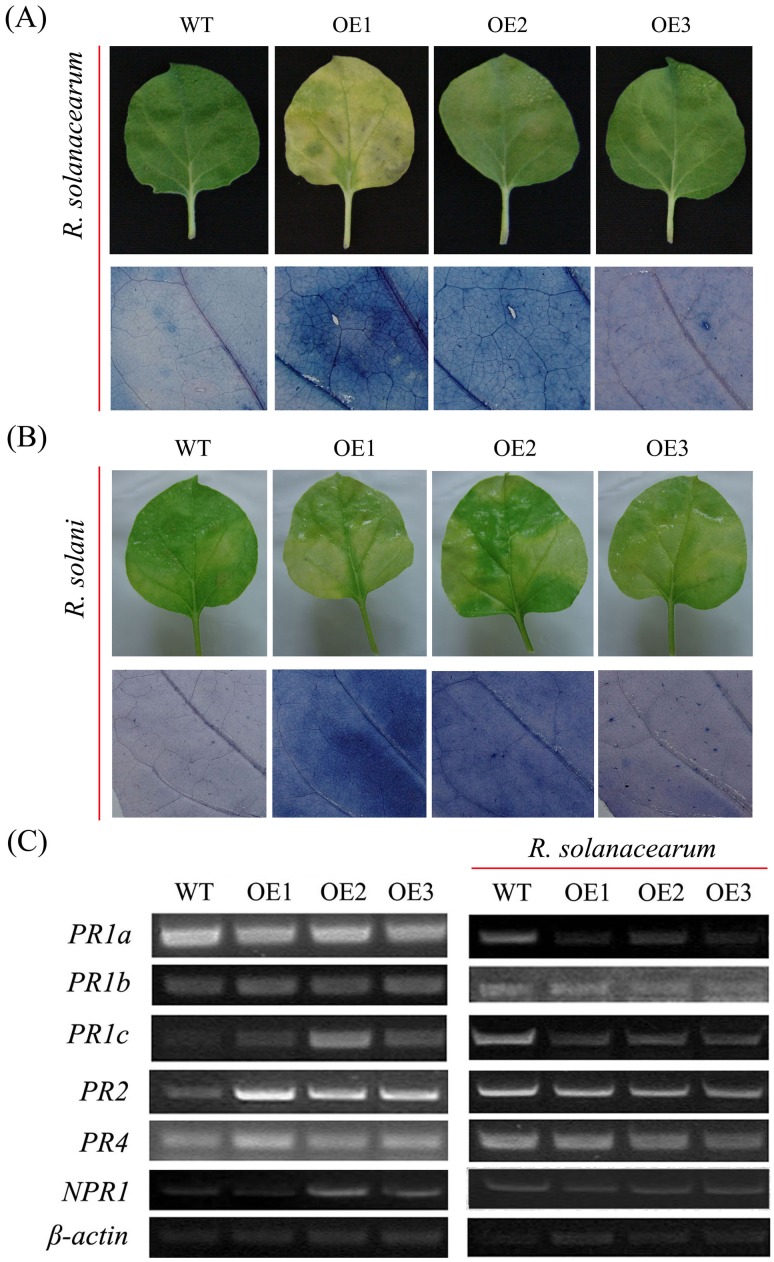
Enhanced susceptibility to *R. solanacearum* and *R. solani* in *GhMKK1*-overexpressing plants. (A) Disease signs on the detached leaves from WT and OE plants 6 d after inoculation with *R. solanacearum* and the relative trypan blue staining, indicating pathogen infection. (B) Disease signs on the detached leaves from WT and OE plants 6 d after inoculation with *R. solani* and the relative trypan blue staining, indicating pathogen infection. (C) RT-PCR analysis of *PR* genes expression in WT and OE plants under normal and *R. solanacearum* inoculation conditions.

To confirm the response of the *GhMKK1*-overexpressing plants to pathogens, 10 µL of an *R. solanacearum* or *R. solani* culture suspension (OD_600_ = 0.6–0.8) was infiltrated into both sides of the main veins of detached *N. benthamiana* leaves using a needleless syringe. Six days after *R. solanacearum* inoculation, the transgenic plants displayed chlorotic signs and enlarged water-soaked lesions on their leaves. However, the wild-type plants showed only mild signs of disease and remained predominantly green. Trypan blue staining of the inoculated leaves revealed relatively more staining in the leaves of the transgenic plants than those of the wild-type plants, indicating that overexpression of *GhMKK1* rendered the plants more susceptible to *R. solanacearum*, including to cell death induced by this organism. Similarly, in the transgenic plants leaves, *R. solani* infection caused larger spots and rapidly spreading chlorosis, and the leaves of these plants exhibited severe wilting and yellowing. In contrast, the spots and chlorosis did not spread in the wild-type plants, resulting in little damage to the leaves. Trypan blue staining resulted in more staining in the leaves of the transgenic plants than in those of the wild-type plants, indicating that overexpression of *GhMKK1* enhanced the plant's susceptibility to *R. solani*, leading to increased levels of *R. solani*-induced cell death in the transgenic plants (Fig. 7A). These results showed that overexpression of *GhMKK1* increased the pathogen susceptibility of the transgenic plants.

To examine the possible mechanisms underlying the enhanced pathogen susceptibility observed in the transgenic plants, we evaluated the expression levels of several disease-responsive genes in *N. benthamiana* including *PR1a*, *PR1b*, *PR1c*, *PR2* (*β-1, 3-glucanase*), *PR4*, and *NPR1*. As shown in Fig. 7C, the expression of *PR1c*, *PR2*, and *NPR1* was constitutively elevated in the transgenic plants compared to the wild-type plants when they were not subjected to any stress treatment. However there was no clear change in the expression levels of *PR1a*, *PR1b*, and *PR4* when no stress was applied to the leaves. Folowing injection with *R. solanacearum*, the expression of *PR1a*, *PR1b*, *PR1c*, and *PR2* decreased in both the wild-type and transgenic plants. In addition, the expression of *PR4* was lower in the transgenic plants than in the wild-type plants following injection (Fig. 7B). The enhanced pathogen susceptibility of the transgenic plants might be due to the reduced expression of these *PR* genes.

## Discussion

The defence mechanisms in plants are sophisticated, including MAPK cascades which transfer singal from sensors to incite cellular responses. As the nodal point of MAPK cascades, the significant role of group A MAPKKs in response to abiotic and biotic stresses have begun to emerge [5], [7], [12], [37]. In the present study, a novel cotton group A MAPKK gene, designated *GhMKK1*, was characterised. Our results revealed that overexpression of *GhMKK1* enhanced salt and drought tolerance and increased the pathogen susceptibility in the transgenic *N. benthamiana*. Notably, *GhMKK1* could either lead to inhibition of ROS production or efficient scavenging of excess ROS. These results not only increase our knowledge of the biological functions of the group A MAPKKs, but also provide new evidence of the role of *GhMKK1* in the regulation of the plant defence responses.

Extracellular signals lead to the activation of MAPK in the cytoplasm, following by its translocation into the nucleus. Accordingly, the definition of MAPKK proteins subcellular localization is so important for exploring the roles of MAPK cascades in signal transduction. Many of the MAPKKs contain a nuclear localization signal peptide (NLS, sequence PKKKRKV) and are localized in the nucleus [38]. Takahashi *et al*. have reported that GFP fluorescence could constitutively observed in the nucleus following the expression of a group D MAPKK in *N. benthamiana* [39]. Similarly, Kong *et al*. suggested that a novel group C MAPKK in maize (*Zea mays*), ZmMKK4, was also localized in the nucleus [6]. In contrast, in the present study, there was no obvious NLS identified in the GhMKK1 amino acid sequence, but the active GhMKK1-GFP was constitutively localized in both the nucleus and the cytoplasm (Fig. 3). Previous studies have suggested that functionally important proteins lacking NLSs are also present in the nucleus [40]. Moreover, several lines of evidence have indicated that MAPKKs may be localized not only in the nucleus, but also in the cytoplasm. For example, AtMKK4 was localized in both of these cellular compartments [41]. Recently, Zhang *et al*. have also reported that a group B MAPKK, ZmMKK3, whose ZmMKK3-GFP fusion protein showed signals throughout the cell, indicating that ZmMKK3 might be localized in both the cytoplasm and the nucleus [42]. These results highlight the importance of controlling MAPK activity at a subcellular level. Therefore, we speculated that GhMKK1 might act as a transcriptional activator in the nucleus, by inducing the expression of a number of target genes and as a signal transmitter in the cytoplasm. The mechanisms underlying the nuclear localization of GhMKK1 and its regulation of downstream targets should be addressed in future work.

Plant cultivation, especially in irrigated lands, is severely affected by salinity stress in many parts of the world. Additionly, drought is also a major constraint to increasing the yields of crop plants. Based on the presence of general and specific abiotic stress tolerance mechanisms, it is reasonable to anticipate that plants exhibit multiple stress perception and signal transduction pathways, which may interact at various steps in their progression. MAPK signalling pathway mediates many stress responses through regulating the expression of various stress-responsive genes [43]. In this study, *GhMKK1* may be involved in the *GhMKK1*-related positive response to abiotic stresses. Firstly, RT-PCR analysis revealed that the expression of *GhMKK1* was strongly induced by various abiotic stresses (Fig. 3). Secondly, the overexpression of *GhMKK1* in *N. benthamiana* enhanced its tolerance to salt and drought stresses, as determined by statistical analyses of germination rates, lateral root length, plant survival rates, and leaf water loss (Fig. 4, 5). In line with these results, the expression and kinase activity of the alfalfa *p44MKK4* gene are activated under cold and drought conditions [44]. *SbMPKK* gene in *Salicornia brachiate* can also possesses a high salt tolerance [45]. Recently, it was reported that OsMKK1, OsMKK4, and OsMKK6 are involved in the salinity and drought stress response of *Oryza. sativa* [46]. However, *Arabidopsis AtMKK9* functions as a negative regulator to participate in the response to salt stress [3], which suggests that the regulatory mechanisms of the response to abiotic stress involving *GhMKK1* significantly vary from those of *AtMKK9*. Even in the same species, MAPKKs functions may be different. Zhang *et al*. reported that *GhMKK5* could induced by salt and dought stress, but transgenic plants overexpressing *GhMKK5* showed reduced salt and drought tolerance [47]. Therefore, elucidating the potential functions of *GhMKK1* is necessary to understand how plants respond to major environmental stressors.

Interestingly, compared with abiotic stress, overexpression of *GhMKK1* increased the pathogen susceptibility of the transgenic plants, and this susceptibility could be determined by the reduced expression of *PR* genes (Fig. 7). These results indicated that *GhMKK1* might be involved in the negative defence responses. Several recent studies have highlighted the importance of MAPKK signaling in the overlapping plant responses to pathogen challenge [48], [49]. But, this defence mechanism may be different. Similar to our result, Brader *et al*. have also reported that overexpression of constitutively active *AtMKK2* increased sensitivity to the fungal necrotroph *Alternaria brassicicola* in OE plant [50]. In contrast, *AtMKK3* can be activated by pathogen and decreased the growth of virulent Pst DC3000 in MKK3-overexpressing plants. This response is most likely mediated through the ability of MKK3 to interact with MPK7, in which MPK7-mediated signaling can activate pathogenesis-related genes in *Arabidopsis* [11]. These results suggest that plant MAPKKs have complex functions, including roles in regulating defence response either positively or negatively.

Environment stress, such as Salinity and drought, disrupt plants photosynthesis and alter the normal homeostasis of cells, resulting in ROS production [12]. Low ROS concentrations can act as secondary messengers regulating cell growth, while high ROS concentrations induce cellular senescence and lead to oxidative damage to special molecules, consequentially injuring tissues [51]. In this study, under drought stress, the overexpression of *GhMKK1* resulted in the decreased accumulation of O_2_^−^ and MDA in transgenic plants compared to wide-type plants. Moreover, there are higher proline concentration and higher antioxidant enzymes activities (CAT, APX, POD and SOD) after drought treatment (Table 3). Consistent with this result, the overexpression of active *AtMKK1* or *AtMKK2* regulates ROS metabolism by controlling the catalase genes that encode the H_2_O_2_-scavenging enzymes [52]. Recently, it was reported that Ca^2+^/CaMs-MKK3-MPK8 pathways are simultaneously involved in the perception of mechanical wounding and in ensuring an optimal balance in the toxic accumulation of ROS in *Arabidopsis* [18]. Therefore, these results indicate that *GhMKK1* might take part in the regulation of the ROS network pathway. Further biochemical and molecular experiments are necessary to elucidate the antioxidant mechanism of *GhMKK1*.

In conclusion, our results strongly suggest that *GhMKK1* is responsive to a variety of stress and that overexpression of *GhMKK1* in *N. benthamiana* plants enhanced their salt and drought tolerance while also increased the transgenic plants' pathogen sensitivity. Furthermore, *GhMKK1*-overexpressing plants exhibited higher antioxidant activity. Although the complex regulatory mechanisms involving *GhMKK1* are still unclear, our results contribute greatly to our understanding of how plants have evolved to cope with environmental stresses. We believe that understanding the specific and shared signal transduction pathways could lead to strategies involving the manipulation of key signaling molecules to achieve broad-spectrum resistance to abiotic stresses and diseases.

## Supporting Information

Table S1 **Gene information in RT-PCR.**
(DOC)

Table S2 **RT-PCR amplification conditions.**
(DOC)
