# Supplementary material for: Cotton GhMKK1 Induces the Tolerance of Salt and Drought Stress, and Mediates Defence Responses to Pathogen Infection in Transgenic Nicotiana benthamiana
Source: PLoS One. 2013 Jul 3;8(7):e68503. doi: 10.1371/journal.pone.0068503 (PMC3700956; doi:10.1371/journal.pone.0068503)
Supplement: Table S1 — Gene information in RT-PCR. (DOC) [file pone.0068503.s001.doc]

**Supplementary Table 1.** Gene information in RT-PCR

| **Name** | **Accession no.** | **Size** | **Primer sequence (5′-3′)** |
| --- | --- | --- | --- |
| *β-actin* | JQ256516.1 | 571 bp | TGGACTCTGGTGATGGTGTC (428-447 bp) forward |
|  |  |  | CCTCCAATCCAAACACTGTA (979-998 bp) reverse |
| *18S rRNA* | U42827.1 | 310 bp | AACTTAAAGGAATTGACGGAAG (1106-1127 bp) forward |
|  |  |  | GCATCACAGACCTGTTATTGCC (1394-1415 bp) reverse |
| *PR1a* | X12485.1 | 286 bp | GGTGTAGAACCTTTGACCTGG (138-158 bp) forward |
|  |  |  | GAACCCTAGCACATCCAACAC (405-423 bp) reverse |
| *PR1b* | X12486.1 | 410 bp | ATCTCACTCTTCTCATGC (27-44 bp) forward |
|  |  |  | TACCTGGAGGATCATAGT (419-436 bp) reverse |
| *PR1c* | X12487.1 | 375 bp | CTTGTCTCTACGCTTCTC (27-44 bp) forward |
|  |  |  | AACACGAACCGAGTTACG (384-401 bp) reverse |
| *PR2* | M60460.1 | 482 bp | ACCATCAGACCAAGATGT (161-178 bp) forward |
|  |  |  | TGGCTAAGAGTGGAAGGT (625-642 bp) reverse |
| *PR4* | EH365959.1 | 217 bp | CAGAACATTAACTGGGATTTGAGAG (161-185 bp) forward |
|  |  |  | CTCCATTTGCTGCATTGATCTACT (355-377 bp) reverse |
| *NPR1* | DQ837218.1 | 551 bp | GCAGCAGACGATGTAATGATGG (685-706 bp) forward |
|  |  |  | TCCACAAGCCTAGTGAGCCTC (1215-1235 bp) reverse |
