# Supplementary material for: Cotton GhMKK1 Induces the Tolerance of Salt and Drought Stress, and Mediates Defence Responses to Pathogen Infection in Transgenic Nicotiana benthamiana
Source: PLoS One. 2013 Jul 3;8(7):e68503. doi: 10.1371/journal.pone.0068503 (PMC3700956; doi:10.1371/journal.pone.0068503)
Supplement: Table S2 — RT-PCR amplification conditions. (DOC) [file pone.0068503.s002.doc]

**Supplementary Table 2.** RT**-**PCR amplification conditions

| **Figure** | **Gene** | **RNA input amount** | **PCR cycle numbers** |
| --- | --- | --- | --- |
| Fig. 4A | *GhMKK1* | 2.0 μg | 32 |
|  | *18S rRNA* | 2.0 μg | 30 |
| Fig. 4B | *GhMKK1* | 2.1 μg | 28 |
|  | *18S rRNA* | 2.1 μg | 26 |
| Fig. 4C | *GhMKK1* | 1.9 μg | 29 |
|  | *18S rRNA* | 1.9 μg | 27 |
| Fig. 4D | *GhMKK1* | 2.0 μg | 30 |
|  | *18S rRNA* | 2.0 μg | 27 |
| Fig. 4E | *GhMKK1* | 1.8 μg | 29 |
|  | *18S rRNA* | 1.8 μg | 26 |
| Fig. 4F | *GhMKK1* | 1.9 μg | 30 |
|  | *18S rRNA* | 1.9 μg | 28 |
| Fig. 4G | *GhMKK1* | 1.8 μg | 27 |
|  | *18S rRNA* | 1.8 μg | 26 |
| Fig. 4H | *GhMKK1* | 1.9 μg | 28 |
|  | *18S rRNA* | 1.9 μg | 26 |
| Fig. 4I | *GhMKK1* | 1.9 μg | 30 |
|  | *18S rRNA* | 1.9 μg | 27 |
| Fig. 4J | *GhMKK1* | 2.0 μg | 29 |
|  | *18S rRNA* | 2.0 μg | 27 |
| Fig. 4K | *GhMKK1* | 2.0 μg | 30 |
|  | *18S rRNA* | 2.0 μg | 28 |
| Fig. 4L | *GhMKK1* | 2.1 μg | 29 |
|  | *18S rRNA* | 2.1 μg | 26 |
| Fig. 5A | *GhMKK1* | 2.5 μg | 28 |
|  | *18S rRNA* | 2.5 μg | 26 |
| Fig. 10C | *PR1a* | 2.0 μg | 32 |
|  | *PR1b* | 2.0 μg | 31 |
|  | *PR1c* | 2.0 μg | 30 |
|  | *PR2* | 2.0 μg | 29 |
|  | *PR4* | 2.0 μg | 31 |
|  | *NPR1* | 2.0 μg | 30 |
|  | *β-actin* | 2.0 μg | 27 |
